# Microbiological Safety and Sensory Quality of Cultivated Mushrooms (*Pleurotus eryngii*, *Pleurotus ostreatus* and *Lentinula edodes*) at Retail Level and Post-Retail Storage

**DOI:** 10.3390/foods10040816

**Published:** 2021-04-09

**Authors:** Simone Schill, Beatrix Stessl, Nadia Meier, Alexander Tichy, Martin Wagner, Martina Ludewig

**Affiliations:** 1 Unit of Food Microbiology, Institute of Food Safety, Food Technology and Veterinary Public Health, University of Veterinary Medicine, 1210 Vienna, Austria; simone_schill@hotmail.com (S.S.); Beatrix.Stessl@vetmeduni.ac.at (B.S.); n-meier@hotmail.com (N.M.); Martin.wagner@vetmeduni.ac.at (M.W.); 2 Austrian Competence Centre for Feed and Food Quality, Safety and Innovation, FFoQSI GmbH, 3430 Tulln an der Donau, Austria; 3 Department of Biomedical Sciences, Bioinformatics and Biostatistics Platform, University of Veterinary Medicine, 1210 Vienna, Austria; Alexander.Tichy@vetmeduni.ac.at

**Keywords:** fresh mushrooms, microbiology contamination, sensory quality, potential foodborne pathogens, storage

## Abstract

In this study, the microbiological and sensory quality of cultivated mushrooms (*Pleurotus ostreatus* and *eryngii* and *Lentinula edodes*) available at the Austrian retail level were determined. Aerobic mesophilic bacteria (AMC), *Enterobacteriaceae* (EB), *Pseudomonadaceae* (PS), lactic acid bacteria (LAB), yeast, moulds and presumptive *Bacillus cereus* were enumerated at the day of purchase and after storage at 4 °C for 7 or 12 days. Additionally, the presence of *Salmonella* spp. and *Listeria monocytogenes* was investigated. Isolates of presumptive spoilage bacteria were confirmed by partial 16S rRNA sequencing. At the day of purchase, 71.2% of the samples were of high microbiological quality and grouped into the low contamination category (AMC < 5.0 log cfu/g), while the sensory quality of 67.1% was categorized as “very good or good”. After storage, the number of samples with high microbial quality was 46.6%, and only 37.0% of the samples scored as “very good or good”. The most abundant species across all mushroom samples were the *Pseudomonas fluorescens* species complex (58.4%) and the potential mushroom pathogen *Ewingella americana* (28.3%). All mushroom samples tested negative for *Salmonella* spp., *L. monocytogenes* and *Bacillus cereus*. The microbiological and sensory quality of the analysed mushrooms at the day of purchase and after storage was considered to be good overall. Longer transport distances were found to have a significant influence on the microbiological and sensory quality.

## 1. Introduction

In the last ten years, global mushroom and truffle production has increased from 7.5 million tonnes (t) in 2009 to 11.8 million t in 2019 [1]. Indeed, a wide range of cultivated mushrooms are available on the global market, the most common being white or champignon de Paris (*Agaricus bisporus*), *Pleurotus* species (*ostreatus, sajor-caju, cystidus, citrinopileatus, flabellatus, eryngii*), shiitake (*Lentinula edodes*) and enoki or winter mushrooms (*Flammulina velutipes*) [2]. In Austria, the self-sufficiency rate for cultivated mushrooms is only 9%. Imported cultivated mushrooms traded in Austrian retail markets mainly originate from farms in Germany, Poland and South Korea [3].

Fresh foods from stable to retail and customers undergo various processes (e.g., harvesting, washing, cutting, packaging, storing and transporting), which may influence the quality and the shelf life [4]. The food industry must ensure food safety and quality standards at all stages of the chain. Nevertheless, foodborne pathogens are found in various foods at European retail level. Mushrooms may also be contaminated with foodborne pathogens, and the Rapid Alert System for Food and Feed (RASFF) of the European Union reported 63 such cases in the period of 1999 to 2020, [5]. The most prevalent zoonotic agents in both dried and brined mushrooms were *Salmonella* spp. (*n* = 24), *Bacillus* (*B.*) *cereus* (*n* = 15), *Clostridium* sulphite reducers/*C. perfringens* (*n* = 13) and in fresh mushrooms *Listeria* (*L.*) *monocytogenes* (*n* = 11). Interestingly, a co-contamination with *Salmonella* and *B. cereus* (≥5.0 log cfu/g) was evident in dried mushrooms (e.g., Mu-Err; *n* = 6 notifications). Messelhäusser et al. reported that 81.5% of dried mushroom samples they tested harboured enterotoxigenic *B. cereus* (≥ 5.0 log cfu/g) [6]. Moreover, a *L. monocytogenes* outbreak was reported by the Centers for Disease Control and Prevention (CDC) and traced back to an enoki mushroom producer in South Korea [7]. Foodborne pathogen contamination occurs either at the mushroom processing plant (from mushroom-growing materials or the food processing environment) [8,9,10,11] or at the retail level [12,13,14].

At retail, the shelf life of food products can be affected by extrinsic factors such as changes in the storage environment (e.g., refrigeration temperature exceeded, moisture level at retail, packing defects) and by intrinsic factors in the product itself (e.g., microbial contamination, especially spoilage bacteria, moisture, pH, redox potential). Mushrooms contain about 80% to 90% water, have an average pH of 6.9 (6.6 to 7.0) and a high CO_2_ respiration rate and are highly perishable in nature [15,16,17]. After harvest, the bacterial load of mushrooms ranges from between 2.5 to 5.8 log cfu/g [18,19]. Both the high water content and microbial load influence the product stability during storage (1.8 to 4.0 log cfu/g increase) [19,20,21]. The sensory deterioration of mushrooms during the post-harvest period, affected by a gradual decrease in moisture as well as intrinsic and bacterial enzyme activities, is associated with discoloration, texture softening and flavour loss [22]. At the retail level, highly variable aerobic mesophilic counts (AMCs) from different cultivated mushrooms have been reported (5.3 to 9.4 log cfu/g) [23]. Here, *Pseudomonadaceae* were the most prevalent group, occurring in all mushroom species, and accounted for the AMC. Average counts of *Enterobacteriaceae* (4.3 log cfu/g) and lactic acid bacteria (1.7 log cfu/g) were much lower and were not present in all mushroom species. Yeasts and moulds were detected in all samples and showed average counts of 3.2 and 3.4 log cfu/g, respectively [23]. Mushroom quality and shelf life depend on various aspects, including raw material quality, the processing environment, and postharvest and storage conditions [22].

In recent studies on the microbiological quality of fresh cultivated mushrooms at the retail level, frequent findings were high counts of aerobic mesophilic bacteria, including *Pseudomonadaceae,* and the presence of *L. monocytogenes* [12,14,23]. However, no studies are available dealing with the interactions of microbial status, sensory quality and spoilage, both in freshly bought and stored mushrooms.

The aim of this study was to determine the potential public health risk of three mushroom species (*Pleurotus ostreatus, Pleurotus eryngii, Lentinula edodes*) available at the Austrian retail level (i) focusing on the microbiological and sensory quality and (ii) evaluating a potential correlation between microbial contamination and sensory decline during storage at 4 °C.

## 2. Materials and Methods

### 2.1. Schematic Overview of the Experimental Program

**Figure 1:**
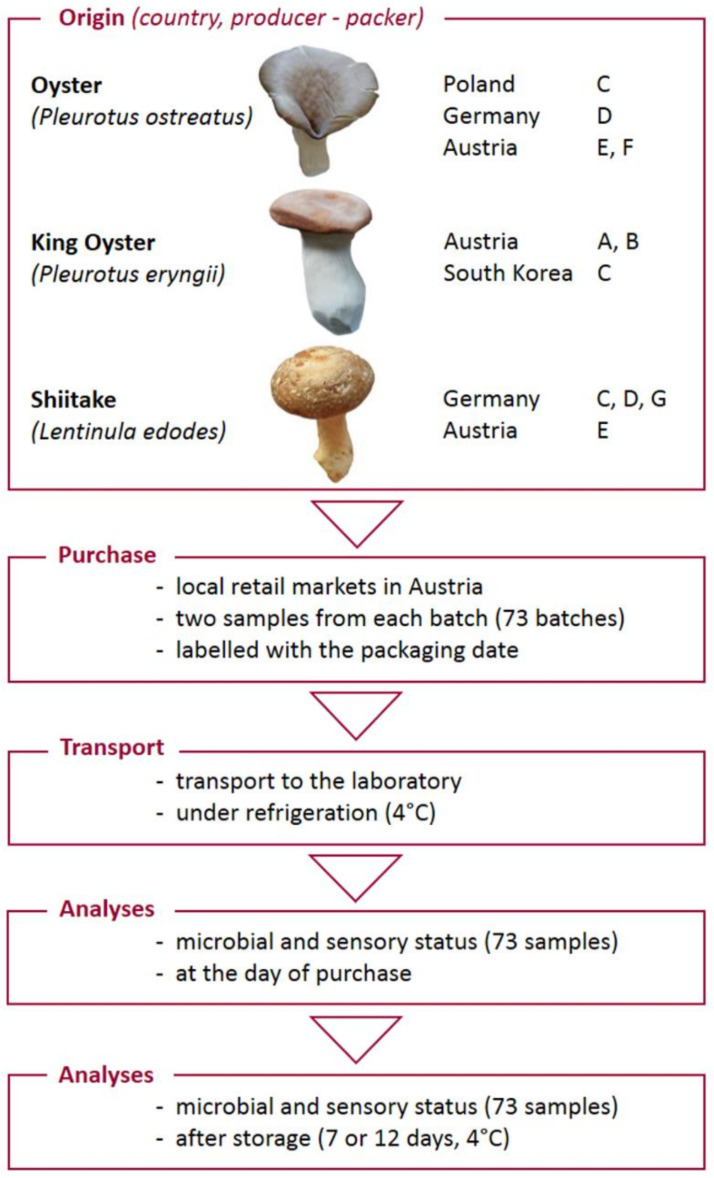
Schematic overview of the experimental program.

The experimental program, comprising the main steps from the origin of cultivated mushroom samples, collection in retail markets, transportation and laboratory analyses, is illustrated in Figure 1. The pilot study primarily evaluated the microbial loads in association with the sensory quality of Austrian-grown and imported fresh cultivated mushrooms at the retail level and the effects of storage. The occurrence of foodborne pathogens in cultivated mushrooms at retail were studied. In single experimental approaches, the microbial status and the sensory quality of each batch were analysed at the day of purhase and after storage.

### 2.2. Collection and Preparation of Mushroom Samples

Oyster, king oyster and shiitake mushrooms (*Pleurotus ostreatus, Pleurotus eryngii, Lentinula edodes)* from six mushroom producers (A, B, D, E, F, G) and one packaging station (C) located in Austria, Germany, Poland and South Korea were investigated (Figure 1, Table 1). Mushroom samples (each 150 to 400 g) were randomly sold at local retailers (*n* = 5) depending on their availability from January 2018 to May 2019. The mushrooms at retail level were either stored at room temperature (2/5 retail markets) or refrigeration temperature (7 °C). In total, 73 mushroom samples were collected in duplicates from the same batch and transported under refrigerated conditions (4 °C). At the day of purchase the initial microbial and sensory status (initial state, IS) of each mushroom sample was determined. At the end of shelf life (after storage at 4°C, AS) the microbial and sensory status was determined for the duplicate sample set (Figure 1). The average storage time for oyster and shiitake mushrooms was seven days, and for king oysters it was 12 days, as the best-before date was defined by a retail chain after the day of packaging.

### 2.3. Microbiological Enumeration, Detection and Isolation

Subsamples (25 g) of each mushroom sample were diluted in 225 mL buffered peptone water (BPW) (Biokar, Groupe Solabia, Pantin Cedex, France) and homogenized for 30 s in a lab blender (Stomacher, Seward Limited, West Sussex, UK). Decimal serial dilutions were prepared up to dilution 10^−7^ in sterile Ringer’s solution (B. Braun, Melsungen, Germany) and plated (each 100 µL) on selective agar media for the enumeration of aerobic mesophilic counts (AMCs), *Enterobacteriaceae* (EB), *Pseudomonadaceae* (PS), lactic acid bacteria (LAB), presumptive *Bacillus cereus* group (BCG) and yeasts and moulds (YM). For dilution 10^−1^ the volume of 1 mL was divided on three agar plates per selective medium.

AMC, EB, PS, LAB, BCG and YM were enumerated on Trypto-Casein Soy Agar with 0.6% yeast extract (TSAYE) (Biokar; ISO 4833-2:2013), Violet Red Bile Glucose (VRBG) Agar (Biokar; ISO 21528-2:2017), Glutamate Starch Phenol-Red (GSP) Agar (Merck KgaA, Darmstadt, Germany), De Man, Rogosa and Sharpe (MRS) Agar (Biokar; ISO 15214:1998), Mannitol Egg Yolk Polymyxin (MYP) Agar (Oxoid Ltd., Hampshire, UK; ISO 7932:2004) and Rose-Bengal Chloramphenicol (RBC) Agar (Oxoid Ltd.) [24,25,26,27]. The selective agar plates were incubated at 25 (PS, YM), 30 (AMC, BCG) and 37 °C (EB, LAB) for 24–48 h (EB, PS and BCG), 72 h (AMC) and 120 h (YM).

All colony morphologies (AMC), typical colonies for hygiene indicators (EB, PS, LAB and YM) and BCG were counted (>10 and <300 colonies for each dilution) and included in the cfu/g calculation. The *Salmonella* spp. initial enrichment was prepared by diluting 25 g in 225 BPW (Biokar), homogenizing for 30 s and incubating at 37 °C for 18 h (ISO 6579-1:2017). The BPW enrichment was transferred each 100 µL and 1 mL to Rappaport-Vassiliadis Soya (RVS; Merck KgaA) and Müller-Kauffmann Tetrathionate-Novobiocin (MKTTn, Merck KgaA) Bouillon and incubated at 42 and 37 °C for 24 h, respectively. *Salmonella* spp. were isolated on Xylose-Lysin-Desoxycholate (XLD) Agar (Merck KgaA) after incubation at 37 °C for 24 h [28].

The *Listeria* spp. and *Listeria monocytogenes* detection was carried out according to ISO 11290-1:2017. From each sample, 25 g was diluted 1:10 in 225 mL Half Fraser broth (HFB; Merck KgaA), homogenized for 30 s and incubated at 30 °C for 24 h. Subsequently, 100 µL HFB was transferred to 10 mL Fraser broth (FB; Merck KgaA) and a loop (10 µL) was streaked on Listeria Agar according to Ottaviani and Agosti (ALOA) (Merck KgaA) and incubated at 37 °C for 24–48 h [29].

Microbial quality of mushrooms before (IS) and after storage was categorised according to the AMC load in five contamination levels: “low”, < 5.0 log cfu/g; “medium”, 5.1–6.5 log cfu/g; “high”, 6.6–8.0 log cfu/g; and “very high”, > 8.1 log cfu/g (according to our internal laboratory standard for fresh products).

### 2.4. Isolate Confirmation and Storage

Generally, typical colonies for hygiene indicator (EB, PS, LAB and BCG) and foodborne pathogenic bacteria (*Salmonella, L. monocytogenes*) were sub-cultivated on TSAYE. The purified isolates (*n* = 413) were stored at −80 °C as 25% glycerol stocks in the strain collection of the Unit of Food Microbiology (University of Veterinary Medicine Vienna). The initial differentiation for hygiene indicators included potassium hydroxide (KOH; 3%; Merck KgaA), catalase (3%; Merck KgaA) and cytochrome-oxidase testing (BioMérieux, Marcy-l’Étoile, France). DNA extraction was performed according to Walsh et al. (1991) [30]. Briefly, isolates were cultivated on TSAYE overnight and incubated at 25 (PS), 30 (BCG) and 37 °C (EB, LAB, *Listeria, Salmonella*). A loop (1 µL) was transferred to 100 µL 0.1 M Tris-HCl buffer (Sigma-Aldrich, St. Louis, MO, USA) vortexed and mixed with 400 µL Chelex^®^ 100 Resin (Bio-Rad, Hercules, CA, USA). The bacterial suspension was heated for 10 min at 100 °C, cooled for 1–2 min and centrifuged at 14,000 rpm for 5 s. The supernatant (100 µL) was transferred to a sterile 1.5 mL Maximum Recovery Eppendorf Tube^®^ (Eppendorf, Hamburg, Germany) and stored at −20 °C.

The bacterial species of hygiene indicator bacteria (AMC, EB, PS), LB and BCG was determined by PCR amplification of the 16S rRNA genes by universal primer 616F (5′-AGAGTTTGATYMTGGCTC-3′) and 1492R (5′-GGYTACCTTGTTACGACTT-3′) (both Microsynth AG, Blagach, Switzerland) [31,32]. The 50 μL PCR reaction mix contained 31.1 µL diethylpyrocarbonate (DEPC) water (Sigma-Aldrich), 5 µL of 10× PCR buffer, 2 µL MgCl_2_ 2 mM, 2.5 µL dNTP mix 250 µM, 2 µL of each primer at 200 nM, 0.4 µL Taq DNA polymerase 5 U/µL (Thermo Fisher Scientific, Waltham, MA, USA) and 5 μL DNA template. The PCR run was performed in a T100 Thermocycler (Bio-Rad, Hercules, CA, USA) with the following conditions: an initial denaturation at 95 °C for 5 min, 30 cycles of denaturation (94 °C for 30 s), primer annealing (52 °C for 30 s) and elongation (72 °C for 60 s) and a final elongation at 72 °C for 7 min. PCR products were electrophoretically separated in a 1.5% agarose gel containing 0.5× Tris-Borate-EDTA (TBE) buffer and 3.5μL peqGREEN DNA gel stain (VWR International, Radnor, PA, USA), at 120 V for 30 min. The DNA standard Thermo Scientific™ GeneRuler™ 100bp (Thermo Fisher Scientific Inc., Waltham, MA, USA) was applied for fragment length comparison.

Partial 16S rRNA gene sequencing was performed by Sanger sequencing technology (LGC Genomics GmbH, Berlin, Germany). The nucleotide sequences were quality evaluated, trimmed via Finch TV 1.4.0 (Geospiza Inc.; https://digitalworldbiology.com/FinchTV) and subsequently compared with sequences from GenBank databases using the Nucleotide BLAST (Basic Local Alignment Search Tool) algorithm from the National Center for Biotechnology Information (https://blast.ncbi.nlm.nih.gov/Blast.cgi) [33]. Sequences were assigned to genus or species level according to best matches and highest similarities (similarity cut-off ≥ 97%). The sequences are provided under the accession number SUB8135232: MT997995-MT998248. Isolates suspicious for *Listeria* and *Salmonella* spp. were confirmed with a species-specific PCR reaction. *Listeria* species differentiation was performed by a multiplex PCR assay targeting the invasion-associated protein (*iap*) gene [34]. *Salmonella* spp. were confirmed by PCR amplification of the invasion protein (*invA*) gene [35].

### 2.5. Sensory Tests

Sensory quality evaluation of mushrooms including appearance, texture and consistency, aroma and taste was performed by three trained panellists [36]. Generally mushroom samples have to be clean, firm, undamaged and largely free from maggot damage, mineral and organic impurities according to Codex Alimentarius Standard, CXS 38-1981 [37]. The typical aroma of oyster mushrooms is described as intense umami and a lightly meaty note and that of king oyster mushrooms is slightly umami [38,39]. Shiitake mushrooms have an intense, sharp and slightly turniplike aroma [40]. Taste was determined by sautéing freshly sliced mushroom cap and stem (10 g) in canola oil (1:10, oil:mushrooms) for five minutes.

The maximum achievable score was five points, which meets the quality standard for edible fungi and fungus products according to Codex Alimentarius Standard [37]. The sensory quality of mushrooms (appearance, consistency and texture, aroma and taste) before and after storage was evaluated according to a grading system 1 to 5 (Supplementary Table S1): 5.0 to 4.0 “very good or good”; 3.9 to 3.0 “satisfactory”; 2.9 to 2.0 “still acceptable” and below 1.9 “not acceptable” and inadequate for human consumption [41].

### 2.6. Statistical Analyses

Descriptive statistics (mean, standard deviation, minimum and maximum values) of variables (AMC, EB, PS, LAB and sensory quality scores) were performed with IBM SPSS V.24 (SPSS Inc., Chicago, IL, USA). Logarithmically transformed microbial counts resulted in normally distributed data and were assessed by Kolmogorov–Smirnov test. Mixed model analysis was used to identify the difference in microbial counts and sensory score between the two time points (initial status and after storage) where producer and country of origin were added as factors to the model. Multiple comparisons were performed using Sidak’s alpha correction procedure. Cross-tabulation table were created to test frequency distributions and associations between categorical variables (microbial counts and sensory quality of initial/stored mushrooms) and were calculated via Pearson’s chi square test. A *p*-value of *p* < 0.05 was considered statistically significant.

## 3. Results

### 3.1. Microbial Counts, Sensory Quality Score and Detection of Foodborne Pathogens

The results of three mushroom species are presented in Table 2. The average counts of AMC, EB and PS (3.8, 2.6 and 3.1 log cfu/g) increased significantly after storage (5.1, 3.3 and 4.0 log cfu/g; *p* < 0.05). Average sensory quality scores decreased in oyster (4.3 to 3.5), king oyster (4.4 to 3.2) and shiitake mushrooms (3.7 to 3.0) significantly after storage (*p* < 0.05). In general, in 60.3% (*n* = 44/73), 47.9% (*n* = 35/73) and 34.2% (*n* = 25/73) of mushroom samples the increase of EB, PS and AMC was ≤ 0.5 log cfu/g after storage. An increase of more than 2.0 log cfu/g for PS, AMC and EB was observed for 31.5% (*n* = 23/73) 28.8% (*n* = 21/73) and 24.6% (*n* = 18/73) of all batches. In particular, a significant increase of AMC and EB counts was observed for king oyster mushrooms after storage (1.9 and 1.3 log cfu/g; *p* < 0.05) (Table 2).

The lowest average AMC (initially: 3.2 log; stored: 4.5 log cfu/g), EB (initially: 1.5 log; stored: 2.5 log cfu/g) and PS counts (initially: 2.4 log; stored: 3.3 log cfu/g) were found in mushrooms produced in Austria. Mushrooms from South Korea were exclusively king oyster. These samples had highest AMC (initially: 5.0 log; stored: 7.6 log cfu/g) and EB counts (initially: 4.7 log; stored: 6.9 log cfu/g). The highest initial PS counts were seen in oyster mushrooms from Poland (4.4 log cfu/g) and after storage in South Korean king oyster mushrooms (5.4 log cfu/g), all from the same packer C.

*Salmonella* spp., *L. monocytogenes*, *Listeria* spp. and presumptive *Bacillus cereus* were not detected in any mushroom sample at the day of purchase or after storage at 4 °C (*n* = 146).

### 3.2. Categorization of Microbiological Contamination and Sensory Quality

**Figure 2:**
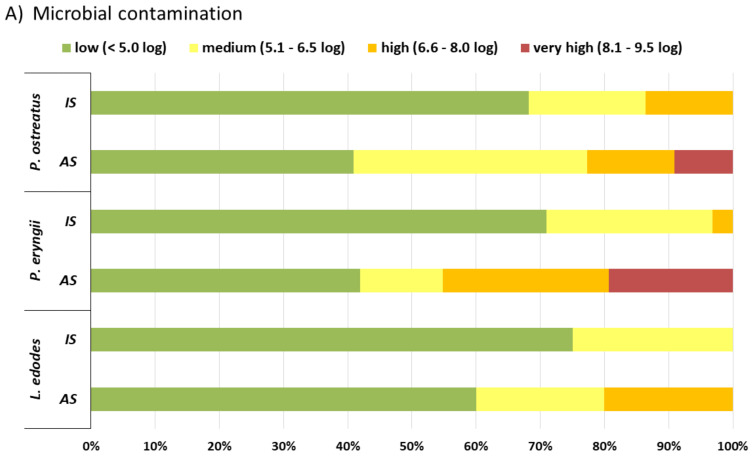

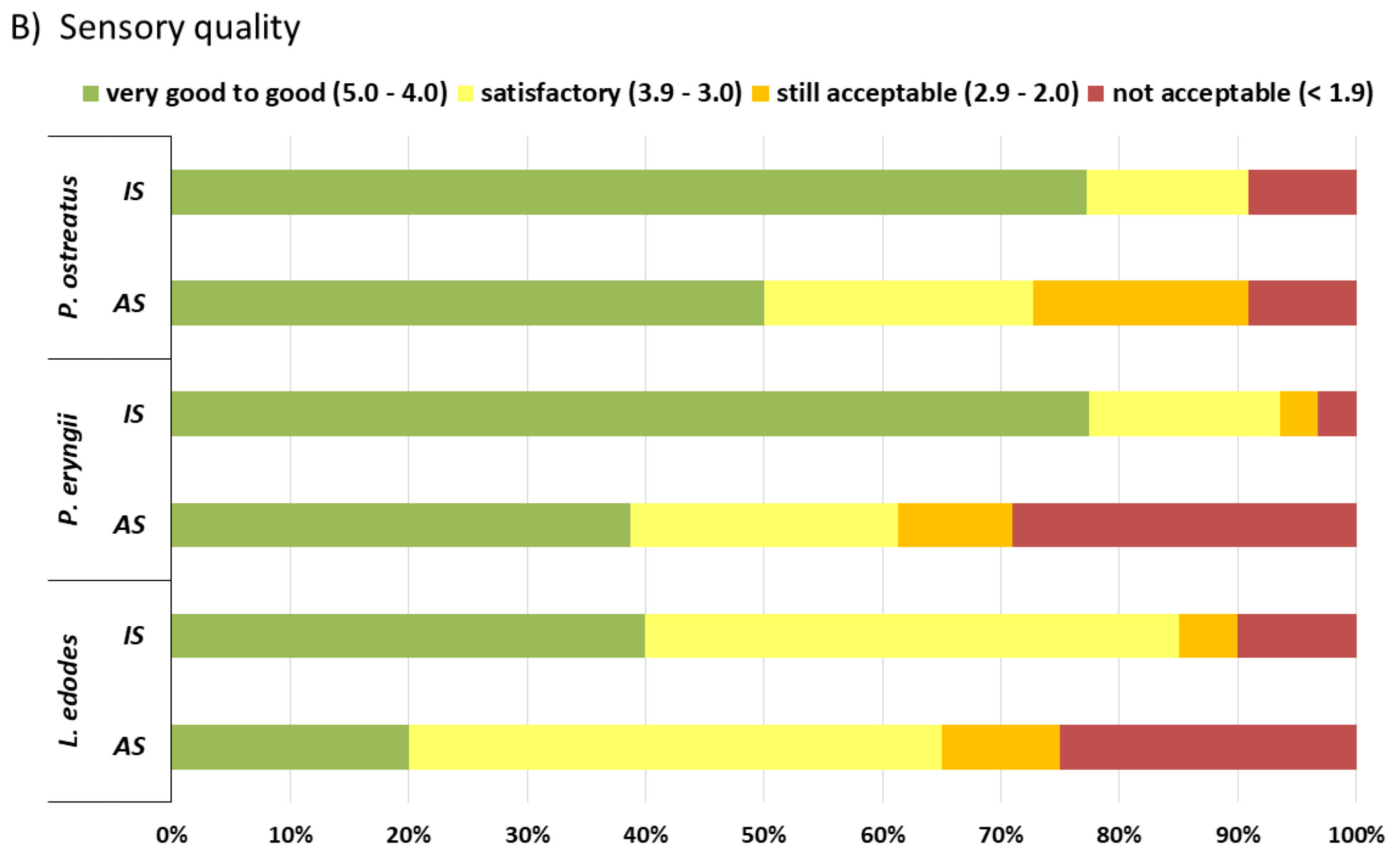
Categorization of microbiological and sensory quality of cultivated mushroom species (**A**,**B**). Percentage (%) of tested samples among several ranges of (**A**) aerobic mesophilic count (AMC) and (**B**) score of sensory quality (QS) analysed on the day of purchase (IS) and after storage (AS).

The level of microbial contamination and sensory quality before (IS) and after storage (AS) is illustrated in Figure 2A,B. The minority of samples (13.6; *n* = 3/22 oyster and 3.2%; *n* = 1/31 king oyster) evidenced a “high” contamination level (AMC; 6.6 to 8.0 log cfu/g) at the day of purchase. After storage, still 40.9% to 60.0% (*n* = 34/73) of mushroom samples were categorized into the contamination level “low” (Figure 2A). Especially, shiitake mushrooms were assigned to level “low” 75.0% (*n* = 15/20) before and 60.0% (*n* = 12/20) after storage. In 9.1% (*n* = 2/22) of oyster and 19.4% (*n* = 6/31) of king oyster samples, microbial load increased to contamination level “very high” (Figure 2A). The cold storage of the mushrooms in the retail shops influenced the level of microbial contamination only in shiitake (contamination level without cooling/with cooling: “low” *n* = 6/21, “medium” *n* = 2/7, “high” *n* = 3/1) (*p* < 0.05).

Initially, the sensory quality of 40.0% to 77.4% (*n* = 49/73) of mushroom samples were scored as “very good or good” (QS: 5.0 to 4.0) and 13.6% to 45.0% (*n* = 17/73) as “satisfactory” (QS: 3.9 to 3.0) (Figure 2B). About, 3.2% to 10.0% of all mushroom species (*n* = 5/73) were evaluated as sensory deficient and grouped to the quality level “not acceptable” (QS < 1.9). Deteriorations in sensory qualities during storage were observed in all mushroom species. Mushroom samples in the category “very good or good” (20.0% to 50.0%, *n* = 27/73) decreased and an increase in the categories “satisfactory” (22.5% to 45.0%, *n* = 21/73) and “still acceptable” (9.0% to 18.1%, *n* = 9/73; QS: 2.9 to 2.0) was observed (Figure 2B). Shiitake mushrooms had the lowest sensory quality because initially only 40.0% (*n* = 8/20) of the samples and after storage 20.0% (*n* = 4/20) could classified as having a “very good or good” sensory quality (Figure 2B).

### 3.3. Isolate Confirmation

**Figure 3:**
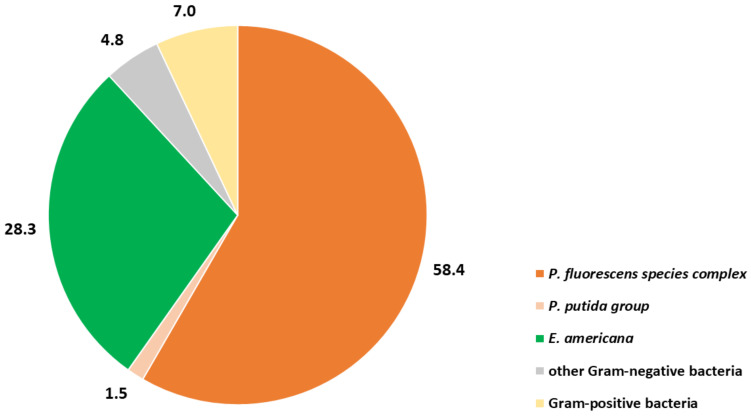
Percentage (%) of bacterial groups among isolates (*n* = 413) originating from cultivated mushroom species.

A total of 413 bacterial isolates were confirmed by partial 16S rRNA gene sequencing. An overview is given in Figure 3. The majority of isolates were accounted to Gram-negative bacteria (93.0%). In detail, 31.7% (*n* = 131/413) and 60.3% (*n* = 249/413) of the isolate set were *Enterobacteriaceae* and *Pseudomonadaceae,* respectively (Figure 3). The most abundant species complex was *P. fluorescens* (58.4% of isolates; *n* = 241/413). The *P. fluorescens* species complex isolates were assigned to following subgroups (SGs): *P. fluorescens* (48.5%; *n* = 117/241), *P. gessardii* (27.8%; *n* = 67/241), *P. mandelii* and *P. koreensis* (each 7.5%; *n* = 18/241), *P. fragi* (5.0%; *n* = 12/241), *P. corrugata* (2.5%; 6/241) and *P. jessenii* (1.2%; 3/241). In the *P. fluorescens* SG, *P. azotoformans* (40.2%; *n* = 47/117), *P. tolaasii* (21.4%; *n* = 25/117), *P. canadensis* (13.7%; *n* = 16/117) and *P. trivialis* (8.5%; *n* = 10/117) were the most abundant species. *P. proteolytica* (50.7%; *n* = 34/67) and *P. brenneri* (46.3%; *n* = 32/67) were the most often isolated members of the *P. gessardii* SG. The most abundant *Enterobacteriaceae* isolates were identified as *E. americana* (89.3%; *n* = 117/131) and *Pantoea beijingensis* (4.6%; *n* = 6/131). The Gram-positive bacteria (7.0%; *n* = 29/413) comprised mostly lactic acid bacteria (e.g., *Lactobacillus sakei, Leuconostoc mesenteroides;* 37.9%; *n* = 11/29) and aerobic spore formers (27.5%; *n* = 8/29). Details are provided in Supplementary Table S2. 

### 3.4. Pseudomonadaceae and Ewingella americana Attribution to Samples and Microbial and Sensory Quality

**Figure 4:**
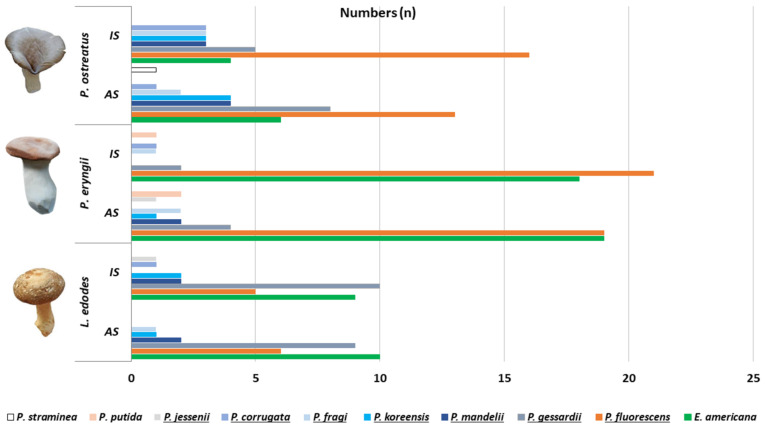
Presence of *Pseudomonas* groups and *Ewingella americana* in *Pleurotus ostreatus, Pleurotus eryngii* and *Lentinula edodes* analysed on the day of purchase (initial state, IS) and after storage (AS). The *Pseudomonas fluorescens* species complex was divided into the subgroups *P. jessenii, corrugata, fragi, koreensis, mandelii, gessardii* and *fluorescens*.

*P. fluorescens* SG and *E. americana* were predominantly isolated from king oyster mushrooms, which can be seen in Figure 4. The most abundant species or bacterial subgroups isolated from all sample types (*n* = 146) were *P. fluorescens* SG (54.7%; *n* = 80), *E. americana* (45.2%; *n* = 66), *P. gessardii* SG (26.0%; *n* = 38) and *P. mandelii* SG (8.9%; *n* = 13) (Figure 4). *P. azotoformans* (17.8%; *n* = 26), *P. proteolytica* (13.6%; *n* = 20), *P. tolaasii* (12.3%; *n* = 18) and *P. brenneri* (11.6%; *n* = 17) were the most observed *Pseudomonas* species detected in the samples.

More sensory deficits (QS ≤ 2.9, “still acceptable”) and higher microbial loads (AMC ≥ 6.6 cfu/g*)* were seen in samples containing *E. americana* and at least one *Pseudomonas* spp. (QS: 46.8%, *n* = 22/47; AMC: 34.0%, *n* = 16/47) compared to samples containing *E. americana* or *Pseudomonas* spp. (QS: 12.3%, *n* = 8/65; AMC: 16.9%, *n* = 11/65, *p* < 0.05). Details can be seen in Table 3. In ten batches of stored oyster and king oyster mushrooms, an increase in the microbial load of ≥ 4.0 log cfu/g was found, most often in samples from packer C and producer A. In all cases, the increase was associated with an increase of PS (*P. fluorescens* SG) or/and EB (*E. americana*) counts.

## 4. Discussion

This study was initiated to evaluate the microbial and sensory quality of fresh cultivated mushrooms sold in the Austrian retail market and to examine the shelf life stability during storage at 4 °C based on the proposed expiration date. The microbiological and sensory quality of three mushroom species (*Pleurotus ostreatus, Pleurotus eryngii, Lentinula edodes*) at the day of purchase and after storage was considered to be good overall. “High” and “very high” levels of contamination was observed in only 8.9% (*n* = 13/146) of mushroom samples, most often in king oyster (11.3%, *n* = 7/62) and oyster (9.1%, *n* = 4/44) mushrooms (Figure 2A). The microbial load of mushroom samples varied widely at the day of purchase and after storage: AMCs at the retail level ranged from log 1.7 to 7.8 at day of purchase and from 1.7 to 9.4 log cfu/g after storage (Table 2). Overall, these findings are in accordance with data from other studies. AMCs at retail level have been reported as follows: 7.7 to 8.4 log cfu/g for champignon, 5.0 to 5.3 log cfu/g for oyster, 4.9 to 6.9 log cfu/g for shiitake and 5.9 log cfu/g for king oyster [23,42,43]. AMCs at the harvest level ranged from 3.1 to 5.8 log cfu/g for champignon, 3.5 log cfu/g for oyster and 4.0 to 4.5 log cfu/g for shiitake [18,20,21,44,45]. Counts of LAB, yeasts and moulds were low (1.1, 2.2 and 2.9 log cfu/g, respectively, Table 2), which is in accordance with findings by Venturini et al. [23]. At the day of purchase, the majority (71.2%) of mushroom samples investigated in our study were categorized as having a “low” level of contamination and similar results were found in the sensory quality, as 67.1% of the samples scored as “very good or good” (Figure 2A,B). After storage, the number of samples with high microbial quality (“low” contamination level) was 46.6% and those with “high” and “very high” contamination increased by 25.9% (Figure 2A). Changes of the visual appearance (cap, stem discoloration or brown or yellow spots, gill sticky, macerated), consistency and texture (soft, rubbery, fibrous), aroma (loss, old, musty, putrid) and taste (loss, bitter) resulted in reduced sensory quality. Only 37.0% of the samples scored as “very good or good”; in total, 21.9% (*n* = 16/73) were deemed “not acceptable”. Most often king oyster (29.0%, *n* = 9/31) and shiitake (25.0%, *n* = 5/20) mushrooms were unfit for consumption (Figure 2B). In contrast to oyster and king oyster, in shiitake mushrooms with sensory deficits a “low” or “medium” microbial contamination was often seen (Figure 2A,B). This observation could be explained by the fact that packed shiitake mushrooms tend to dry out during storage. Other studies reported an AMC increase in shiitake mushrooms during storage at 4 °C for six to eight days by 2.0–3.0 log cfu/g as well as a deterioration of the sensory quality [20,21,45].

In our study high AMCs were associated with the presence of *Pseudomonas* spp. and *E. americana.* This is consistent with the literature [18,23,43,46]. The majority of *Pseudomonas* isolates were assigned to *P. fluorescens* (54.7%) and *P. gessardii* (26.0%) subgroups (Figure 4). In particular, *P. azotoformans*, *P. proteolytica*, *P. tolaasii* and *P. brenneri* were often detected. Frequently, bacterial mushroom spoilage is associated with *P. tolaasii*, leading to brown blotches and the resulting yellow to dark brown lesions on the mushroom cap, which are induced by the toxin tolaasin [47,48,49]. Additionally, other bacteria such as *P. azotoformans* and *P. brenneri* are reported to cause spoilage of diseased mushrooms [50]. In industrial cultivation, other bacteria such as *P. azotoformans, P. fluorescens*, *P. putida* and *Bacillus* spp. are used as antagonists that suppress the aforementioned mushroom pathogenic bacteria [51,52,53]. This could explain why these bacteria were seen in our samples as well. *E. americana* was found in 45.2% of the samples and was present in all mushroom species (Figure 4). This is in accordance with Reyes et al. (2004) where *E. americana* was highly prevalent in oyster (76.7%) and shiitake mushrooms (73.3%) purchased at retail markets [43]. *E. americana* has been reported to be an opportunistic pathogen in humans, isolated in patients with immunosuppressed status [54]. According to the literature, *E. americana* might either cause internal stipe necrosis in champignon and king oyster mushrooms, or soft rot and mild browning in oyster mushrooms. Further, the bacteria is a “commensal” in healthy champignon, oyster and shiitake mushrooms [43,55,56]. Recent results show that blotch symptoms of champignon are often associated with bacteria of the *Enterobacteriaceae* family, while *Pseudomonadaceae* were present in healthy and diseased mushrooms [57]. In our study, in stored mushroom samples with increased *Enterobacteriaceae* counts *E. americana* was dominant (83.3%; *n* = 15/18). We found that samples containing *E. americana* and at least one *Pseudomonas* species more often had sensory deficits and higher microbial loads (*p* < 0.05) (Table 3). The bacterial community of samples classified as unfit for consumption (OS < 1.9) contained high levels of *E. americana* and diverse *Pseudomonas* species (76.2%; *n* = 16/21) including potential mushroom pathogens (*P. azotoformans*, *P. brenneri* and *P. tolaasii*). Three samples (two king oyster and one oyster) also showed symptoms as described by Gonzales et al. (2012) [56]. It seems that the co-occurrence of *E. americana* and *Pseudomonas* species in one sample influences the storage stability of mushrooms.

*L. monocytogenes*, *Salmonella* spp. and presumptive *B. cereus* were not detected in any of our samples. A Spanish study was also unable to isolate *L. monocytogenes* and *Salmonella* spp. in cultivated mushrooms [23]. However, in oyster, king oyster and shiitake mushrooms sold in Chinese markets, *L. monocytogenes* was found in 6.7%, 4.4% and 2.9% of the samples, respectively [12]. Recently, two studies reported an incidence of *L. monocytogenes* of 0.8% [14] and 50.0% [58] in processed mushrooms. There is increasing evidence that *L. monocytogenes* results from contamination during processing. The pathogen has many stress adaptions that enable survival in a wide range of environmental niches [59,60]. Further, *L. monocytogenes* has also been isolated from the mushroom farm environment, emphasizing the importance of monitoring the production chain from the substrate production to harvest, processing and packaging [8,10,61].

## 5. Conclusions

This study focused on the quality of fresh mushrooms at the retail level, analysed on the day of purchase and after storage. The majority of mushrooms investigated at the day of purchase had a high microbiological quality. After storage, the AMC increased, especially in oyster and the South Korean king oyster mushrooms. The sensory quality of mushrooms did not always change depending on the microbiological load. Changes in consistency and texture, including drying and increased toughness, occurred during storage, and might have been influenced by the type of packaging. Poor hygiene facility management, re-packaging and long transport distances might lead to product spoilage and reduced shelf life. The king oyster mushrooms from producer B had the highest product stability and the shortest transport distances to the supermarket compared to packer C, which re-packed the mushrooms and had the longest transport distances. Severe changes of the sensory quality were mainly observed in such stored samples where *E. americana* were found in combination with different *Pseudomonas* species. Therefore, the monitoring of these bacteria species could be useful in the assessment of the shelf life. It is known that the stability of mushrooms during storage is influenced by many interacting factors. To obtain microbiologically safe mushrooms and maintain their sensory quality, further research should therefore focus on various steps along the processing chain and on the effectiveness of packaging systems to prevent moisture loss and microbial growth.

## Figures and Tables

**Table 1:** Sample description of cultivated mushroom species included in this study.

Mushroom Species ^a^	Country of Origin	Producer/Packer ^c^	Samples (%) ^b^
*Pleurotus ostreatus*	Poland	C ^c^	12 (8.2)
(Oyster mushroom)	Germany	D	12 (8.2)
	Austria	E	16 (11.0)
	Austria	F	4 (2.7)
*Pleurotus eryngii*	Austria	A	16 (11.0)
(King oyster mushroom)	Austria	B	26 (17.8)
	South Korea	C ^c^	20 (13.7)
*Lentinula edodes*	Germany	C^c^	2 (1.4)
(Shiitake mushroom)	Germany	D	12 (8.2)
	Germany	G	4 (2.7)
	Austria	E	22 (15.1)
Total number of samples			146 (100.0)

Abbreviations: ^a^ Species specific name, common species name in brackets; ^b^ Number of samples and percentage of the total; ^c^ Mushrooms distributed by packer.

**Table 2:** Average microbial counts and sensory quality scores of cultivated mushroom species on the day of purchase (initial) and after storage.

Mushroom Species	ID	AMC	EB	PS	Moulds	Yeasts	LAB	BC	QS
*Pleurotus ostreatus*	IS	4.2 ± 1.7 ^a^ (1.7–7.6) ^b^	1.9 ± 1.7 (1.0–6.9)	3.7 ± 2.0 (1.0–7.6)	2.5 ± 1.3 (1.0–4.6)	2.4 ± 1.4 (1.0–5.1)	1.0 ± 0.0 (1.0–1.0)	<1.0	4.3 ± 1.0 (1.3–5.0)
	AS	5.2 ± 1.8 (1.7–8.6)	2.6 ± 2.3 (1.0–7.2)	4.9 ± 1.9 (1.0–8.5)	3.1 ± 1.1 (1.0–4.8)	3.3 ± 1.6 (1.0–5.4)	1.0 ± 0.0 (1.0–1.0)	<1.0	3.5 ± 1.2 ^c^ (0.9–5.0)
*Pleurotus eryngii*	IS	3.7 ± 1.6 (1.7–7.8)	3.0 ± 1.9 (1.0–7.6)	2.7 ± 1.8 (1.0–7.0)	3.2 ± 1.0 (1.0–5.2)	1.4 ± 1.2 (1.0–6.6)	1.2 ± 0.85 (1.0–5.4)	<1.0	4.4 ± 0.8 (1.4–5.0)
	AS	5.6 ± 2.6 ^c^ (1.7–9.4)	4.2 ± 2.8 ^c^ (1.0–9.0)	3.6 ± 3.1 (1.0–8.8)	3.0 ± 1.3 (1.0–5.2)	1.9 ± 1.8 (1.0–8.0)	1.3 ± 1.0 (1.0–5.7)	<1.0	3.2 ± 1.3 ^c^ (1.1–5.0)
*Lentinula edodes*	IS	3.7 ± 1.5 (1.7–6.3)	2.6 ± 1.8 (1.0–6.3)	3.0 ± 1.9 (1.0–5.8)	2.5 ± 0.9 (1.0–4.4)	2.4 ± 1.5 (1.0–5.5)	1.2 ± 0.7 (1.0–4.0)	<1.0	3.7 ± 1.0 (1.2–5.0)
	AS	4.3 ± 1.8 (1.7–6.7)	2.7 ± 2.0 (1.0–6.6)	3.5 ± 2.2 (1.0–6.7)	3.0 ± 1.1 (1.0–5.2)	2.4 ± 1.5 (1.0–5.4)	1.0 ± 0.0 (1.0–1.0)	<1.0	3.0 ± 1.1 ^c^ (1.0–4.7)

Abbreviations: ID—day of investigation; IS—initial state (day of purchase); AS—after storage at 4 °C for seven days (oyster and shiitake) or 12 days (king oyster); AMC—aerobic mesophilic count; EB—*Enterobacteriaceae*; PS—*Pseudomonadaceae*; LAB—lactic acid bacteria; BC—*Bacillus cereus* (< 1.0, below the limit of detection); QS—score of sensory quality. Mean microbial counts are calculated in log cfu/g. ^a^ Mean ± SD; ^b^ Minimum–Maximum; ^c^ Mean values differ significantly (*p* < 0.05) from the day of purchase.

**Table 3:** Occurrence of *Ewingella americana* and *Pseudomonadaceae* relative to the sensory quality and aerobic mesophilic counts.

Species	Number of Samples	Number of Samples (%)	
		QS ≤ 2.9 ^a^	AMC ≥ 6.6 cfu/g ^b^
*E. americana*	19	2 (10.5)	4 (21.1)
*E. americana*^c^ + *P. azotoformans* + *P. brenneri* + *P. tolaasii*	30	15 (50.0)	10 (33.3)
*E. americana*^c^ + other *P*. species	17	7 (41.2)	6 (35.3)
*P. azotoformans* + *P. brenneri* + *P. tolaasii*^d^	24	2 (8.3)	5 (20.8)
other *P*. species ^d^	22	4 (18.2)	2 (9.1)

Abbreviations: ^a^ QS—score of sensory quality, “still acceptable”; ^b^ AMC—aerobic mesophilic count, “high” and “very high” microbial contamination; ^c^ *Ewingella* (*E.*) *americana* in co-occurrence with at least one of the listed *Pseudomonas* (*P.*) spp.; ^d^ one or more of the listed *P.* spp.

## Data Availability

There are no further data available.

## Supplementary Materials

The following are available online at https://www.mdpi.com/article/10.3390/foods10040816/s1, Table S1: Defined sensory deficits based on quality characteristics of cultivated mushroom species, Table S2: Description of isolates detected in oyster, king oyster and shiitake mushrooms at initial status and after storage.
