# Supplementary material for: Microbiological Safety and Sensory Quality of Cultivated Mushrooms (Pleurotus eryngii, Pleurotus ostreatus and Lentinula edodes) at Retail Level and Post-Retail Storage
Source: Foods. 2021 Apr 9;10(4):816. doi: 10.3390/foods10040816 (PMC8070540; doi:10.3390/foods10040816)
Supplement: Supplementary file 1 [file foods-10-00816-s001.pdf]

Supplementary Table 1. Defined sensory deficits based on quality characteristics of cultivated mushroom species.

Quality characteristic	Sensory deficits				Grades – quality score [a]
	Mushroom batch	Cap	Stem	Gills	
**Appearance and colour in raw and non-processed state**	mineral impurities<br>organic impurities<br>maggot damage<br>overripe	discolouration<br>surface moist/sticky<br>brown spots<br>yellow spots<br>mould	discolouration<br>surface moist/sticky<br>brown spots<br>yellow spots	discolouration<br>sticky<br>macerated	1 – 5
**Consistency and texture**	soft<br>external moisture<br>dry	soft<br>floury<br>spongy	diminished elasticity<br>rubbery<br>fibrous		1 – 5
**Consistency and texture after cooking**	rubbery<br>tough<br>fibrous				1 – 5
**Aroma**	loss of typical aroma<br>old<br>foreign	musty<br>putrid<br>phenolic			1 – 5
**Taste after cooking**	loss of typical taste<br>bitter<br>astringent<br>sweet				1 – 5

Supplementary Table 2. Description of isolates detected in oyster, king oyster and shiitake mushrooms at initial status and after storage.

Strain-ID	Isolation-Source	Origin	Producer	n	Group	Description
KS1	King oyster	Austria	B	1	CORRUG	*P. brassicacearum* subsp. *neoaurantiaca*
KS12	King oyster	Austria	B	1	FLUOR	*P. azotoformans*
KS257	King oyster	Austria	A	2	FLUOR	*P. azotoformans*
KS259	King oyster	Austria	A	2	FRAGI	*P. deceptionensis*
KS261	King oyster	Austria	A	1	FLUOR	*P. tolaasii*
KS262	King oyster	Austria	A	3	PUT	*P. plecoglossicida*
KS269	King oyster	Austria	A	1	FLUOR	*P. tolaasii*
KS274	King oyster	Austria	A	4	FLUOR	*P. azotoformans*
KS275	King oyster	Austria	A	1	MAND	*P. lini*
KS422	King oyster	Austria	A	2	FLUOR	*P. azotoformans*
KS424	King oyster	Austria	A	2	KORE	*P. koreensis*
AU414	Oyster	Austria	E	2	FLUOR	*P. lurida*
KS420	King oyster	South Korea	C	1	FLUOR	*P. canadensis*
KS421	King oyster	South Korea	C	1	FLUOR	*P. azotoformans*
KS426	King oyster	South Korea	C	2	FLUOR	*P. canadensis*
AU432	Oyster	Poland	C	2	FLUOR	*P. tolaasii*
AU438	Oyster	Poland	C	1	CORRUG	*P. frederiksbergensis*
AU439	Oyster	Poland	C	1	FLUOR	*P. tolaasii*
AU448	Oyster	Austria	E	2	KORE	*P. koreensis*
AU457	Oyster	Austria	E	2	KORE	*P. koreensis*
SH455	Shiitake	Austria	E	1	KORE	*P. koreensis*
KS494	King oyster	Austria	A	1	FLUOR	*P. azotoformans*
KS495	King oyster	Austria	A	1	GESS	*P. proteolytica*
KS521	King oyster	Austria	A	1	FLUOR	*P. marginalis*
KS522	King oyster	Austria	A	1	FLUOR	*P. azotoformans*
KS523	King oyster	Austria	A	1	MAND	*P. prosekii*
KS524	King oyster	Austria	A	1	JESS	*P. umsongensis*
KS525	King oyster	Austria	A	2	PUT	*P. plecoglossicida*
KS550	King oyster	Austria	A	1	PUT	*P. putida*

KS552	King oyster	Austria	A	2	GESS	*P. proteolytica*
AU469	Oyster	Austria	E	2	FLUOR	*P. azotoformans*
AU499	Oyster	Austria	E	2	FLUOR	*P. fluorescens*
KS475	King oyster	Austria	B	2	FLUOR	*P. tolaasii*
SH480	Shiitake	Germany	G	2	GESS	*P. proteolytica*
SH486	Shiitake	Germany	G	2	FLUOR	*P. poae*
SH488	Shiitake	Germany	G	1	FRAGI	*P. endophytica*
KS504	King oyster	South Korea	C	2	FLUOR	*P. canadensis*
KS537	King oyster	South Korea	C	1	FLUOR	*P. trivialis*
KS538	King oyster	South Korea	C	1	FLUOR	*P. trivialis*
KS510	King oyster	Austria	A	2	FLUOR	*P. azotoformans*
KS544	King oyster	Austria	A	3	FLUOR	*P. azotoformans*
KS545	King oyster	Austria	A	1	GESS	*P. brenneri*
AU514	Oyster	Austria	E	1	FLUOR	*P. fluorescens*
AU516	Oyster	Austria	E	2	GESS	*P. proteolytica*
KS533	King oyster	South Korea	C	1	FLUOR	*P. tolaasii*
KS534	King oyster	South Korea	C	1	FLUOR	*P. canadensis*
KS562	King oyster	South Korea	C	2	FLUOR	*P. azotoformans*
KS527	King oyster	Austria	A	2	FLUOR	*P. azotoformans*
KS570	King oyster	Austria	A	2	FLUOR	*P. azotoformans*
SH554	Shiitake	Austria	E	2	FLUOR	*P. tolaasii*
KS566	King oyster	South Korea	C	1	GESS	*P. proteolytica*
KS584	King oyster	South Korea	C	2	FRAGI	*P. helleri*
KS598	King oyster	South Korea	C	1	FLUOR	*P. canadensis*
KS599	King oyster	South Korea	C	1	GESS	*P. proteolytica*
KS576	King oyster	South Korea	C	1	FLUOR	*P. tolaasii*
KS577	King oyster	South Korea	C	1	FLUOR	*P. canadensis*
KS592	King oyster	South Korea	C	1	FLUOR	*P. trivialis*
KS612	King oyster	South Korea	C	2	FLUOR	*P. tolaasii*
KS644	King oyster	Austria	A	1	GESS	*P. brenneri*
KS645	King oyster	Austria	A	1	FRAGI	*P. helleri*
KS1060	King oyster	Austria	A	1	FLUOR	*P. tolaasii*
KS656	King oyster	South Korea	C	1	FLUOR	*P. trivialis*
KS657	King oyster	South Korea	C	1	FLUOR	*P. fluorescens*

KS704	King oyster	South Korea	C	1	FLUOR	*P. tolaasii*
AU670	Oyster	Austria	F	2	FLUOR	*P. azotoformans*
AU672	Oyster	Austria	F	2	FRAGI	*P. helleri*
AU691	Oyster	Austria	F	2	FLUOR	*P. azotoformans*
AU698	Oyster	Austria	F	1	KORE	*P. koreensis*
AU719	Oyster	Poland	C	2	GESS	*P. brenneri*
AU720	Oyster	Poland	C	2	FLUOR	*P. poae*
AU724	Oyster	Poland	C	2	STRAM	*P. punonensis*
AU726	Oyster	Poland	C	1	KORE	*P. helmanticensis*
AU747	Oyster	Poland	C	2	FLUOR	*P. azotoformans*
AU751	Oyster	Poland	C	1	FRAGI	*P. helleri*
AU752	Oyster	Poland	C	1	FRAGI	*P. versuta*
AU739	Oyster	Poland	C	2	FLUOR	*P. tolaasii*
KS767	King oyster	South Korea	C	1	FLUOR	*P. trivialis*
KS768	King oyster	South Korea	C	1	FLUOR	*P. fluorescens*
KS762	King oyster	Austria	B	1	FLUOR	*P. azotoformans*
AU776	Oyster	Austria	E	1	FLUOR	*P. trivialis*
AU815	Oyster	Austria	E	2	GESS	*P. brenneri*
AU816	Oyster	Austria	E	2	GESS	*P. proteolytica*
SH757	Shiitake	Germany	D	2	GESS	*P. proteolytica*
SH778	Shiitake	Germany	D	2	GESS	*P. brenneri*
KS785	King oyster	South Korea	C	2	FLUOR	*P. azotoformans*
KS798	King oyster	South Korea	C	2	FLUOR	*P. canadensis*
SH806	Shiitake	Germany	D	1	MAND	*P. lini*
SH819	Shiitake	Germany	D	2	FLUOR	*P. azotoformans*
SH822	Shiitake	Germany	D	2	GESS	*P. brenneri*
SH833	Shiitake	Germany	D	2	FLUOR	*P. azotoformans*
KS812	King oyster	Austria	B	1	FLUOR	*P. canadensis*
KS813	King oyster	Austria	B	1	FLUOR	*P. azotoformans*
SH835	Shiitake	Austria	E	2	FLUOR	*P. trivialis*
AU841	Oyster	Austria	E	1	FLUOR	*P. trivialis*
AU842	Oyster	Austria	E	2	GESS	*P. proteolytica*
AU849	Oyster	Austria	E	2	GESS	*P. brenneri*
AU850	Oyster	Austria	E	2	GESS	*P. proteolytica*

SH875	Shiitake	Austria	E	2	GESS	*P. proteolytica*
SH879	Shiitake	Austria	E	1	KORE	*P. koreensis*
AU880	Oyster	Poland	C	2	MAND	*P. silesiensis*
AU882	Oyster	Poland	C	1	CORRU G	*P. corrugata*
AU905	Oyster	Poland	C	1	FLUOR	*P. tolaasii*
AU883	Oyster	Poland	C	2	MAND	*P. silesiensis*
AU886	Oyster	Poland	C	1	CORRU G	*P. corrugata*
SH888	Shiitake	Austria	E	2	GESS	*P. proteolytica*
SH890	Shiitake	Austria	E	2	GESS	*P. proteolytica*
AU898	Oyster	Germany	D	1	KORE	*P. koreensis*
AU899	Oyster	Germany	D	2	GESS	*P. proteolytica*
AU901	Oyster	Germany	D	2	FLUOR	*P. azotoformans*
AU907	Oyster	Germany	D	1	GESS	*P. brenneri*
SH910	Shiitake	Austria	E	2	GESS	*P. proteolytica*
SH919	Shiitake	Austria	E	2	KORE	*P. helmanticensis*
SH921	Shiitake	Austria	E	2	FLUOR	*P. azotoformans*
AU912	Oyster	Germany	D	2	FLUOR	*P. canadensis*
AU924	Oyster	Germany	D	1	FLUOR	*P. trivialis*
AU926	Oyster	Germany	D	1	GESS	*P. brenneri*
AU966	Oyster	Germany	D	1	FLUOR	*P. marginalis*
SH930	Shiitake	Germany	D	2	GESS	*P. brenneri*
SH940	Shiitake	Germany	D	3	GESS	*P. brenneri*
AU934	Oyster	Austria	F	2	FLUOR	*P. tolaasii*
AU937	Oyster	Austria	F	1	FLUOR	*P. canadensis*
AU972	Oyster	Austria	F	1	FLUOR	*P. azotoformans*
AU974	Oyster	Austria	F	1	FLUOR	*P. tolaasii*
AU976	Oyster	Poland	C	1	CORRU G	*P. corrugata*
AU944	Oyster	Poland	C	2	KORE	*P. helmanticensis*
SH979	Shiitake	Germany	D	1	FLUOR	*P. canadensis*
SH982	Shiitake	Germany	D	1	GESS	*P. brenneri*
SH951	Shiitake	Germany	D	1	GESS	*P. proteolytica*
SH962	Shiitake	Germany	D	1	MAND	*P. lini*

AU983	Oyster	Germany	D	2	FLUOR	*P. poae*
AU986	Oyster	Germany	D	1	GESS	*P. proteolytica*
AU955	Oyster	Germany	D	2	FRAGI	*P. fragi*
AU956	Oyster	Germany	D	1	FLUOR	*P. azotoformans*
AU957	Oyster	Germany	D	1	MAND	*P. lini*
AU960	Oyster	Austria	E	1	FLUOR	*P. canadensis*
AU991	Oyster	Austria	E	2	GESS	*P. brenneri*
SH1001	Shiitake	Austria	E	2	GESS	*P. proteolytica*
SH1007	Shiitake	Germany	C	1	GESS	*P. proteolytica*
SH995	Shiitake	Germany	C	2	GESS	*P. proteolytica*
SH997	Shiitake	Germany	C	2	FLUOR	*P. tolaasii*
AU1009	Oyster	Poland	C	1	KORE	*P. helmanticensis*
AU1010	Oyster	Poland	C	1	MAND	*P. silesiensis*
AU1011	Oyster	Poland	C	1	CORRUG	*P. corrugata*
AU1012	Oyster	Poland	C	1	FLUOR	*P. synxantha*
AU1024	Oyster	Poland	C	2	KORE	*P. helmanticensis*
AU1026	Oyster	Poland	C	2	MAND	*P. silesiensis*
SH1014	Shiitake	Germany	D	3	GESS	*P. brenneri*
SH1016	Shiitake	Germany	D	1	FLUOR	*P. orientalis*
SH1019	Shiitake	Germany	D	1	MAND	*P. lini*
SH1030	Shiitake	Germany	D	2	GESS	*P. brenneri*
SH1033	Shiitake	Germany	D	2	MAND	*P. lini*
AU1022	Oyster	Germany	D	2	FLUOR	*P. azotoformans*
AU1036	Oyster	Germany	D	2	GESS	*P. brenneri*
AU1037	Oyster	Germany	D	1	FLUOR	*P. poae*
SH1041	Shiitake	Germany	D	2	JESS	*P. reinekei*
SH1045	Shiitake	Germany	D	1	CORRUG	*P. brassicacearum subsp. neoaurantiaca*
SH1046	Shiitake	Germany	D	1	FLUOR	*P. tolaasii*
SH1052	Shiitake	Germany	D	3	GESS	*P. brenneri*
AU1055	Oyster	Austria	E	1	GESS	*P. gessardii*
AU1028	Oyster	Poland	C	1	STEN	*Stenotrophomonas rhizophila*
SH789	Shiitake	Germany	D	2	FLUOR	*P. synxantha*

AU987	Oyster	Austria	E	2	MAND	*P. lini*
KS2	King oyster	Austria	B	2	EB	*Ewingella americana*
KS11	King oyster	Austria	B	1	EB	*Ewingella americana*
KS18	King oyster	Austria	B	2	EB	*Ewingella americana*
KS21	King oyster	Austria	B	1	EB	*Kluyvera cryocrescens*
KS418	King oyster	South Korea	C	2	EB	*Ewingella americana*
KS428	King oyster	South Korea	C	2	EB	*Ewingella americana*
AU430	Oyster	Poland	C	2	EB	*Rahnella aquatilis*
AU440	Oyster	Poland	C	2	EB	*Ewingella americana*
KS434	King oyster	Austria	B	2	EB	*Ewingella americana*
KS467	King oyster	Austria	B	2	EB	*Ewingella americana*
KS450	King oyster	Austria	B	2	EB	*Ewingella americana*
KS492	King oyster	Austria	A	2	EB	*Ewingella americana*
KS519	King oyster	Austria	A	2	EB	*Ewingella americana*
SH476	Shiitake	Germany	G	2	EB	*Ewingella americana*
SH484	Shiitake	Germany	G	2	EB	*Ewingella americana*
KS502	King oyster	South Korea	C	2	EB	*Ewingella americana*
KS536	King oyster	South Korea	C	1	EB	*Ewingella americana*
KS539	King oyster	South Korea	C	1	EB	*Pantoea beijingensis*
KS508	King oyster	Austria	A	1	EB	*Ewingella americana*
KS512	King oyster	Austria	A	1	EB	*Acinetobacter guillouiae*
KS542	King oyster	Austria	A	2	EB	*Ewingella americana*
KS529	King oyster	South Korea	C	2	EB	*Ewingella americana*
KS560	King oyster	South Korea	C	2	EB	*Pantoea beijingensis*
SH558	Shiitake	Austria	E	2	EB	*Ewingella americana*
KS564	King oyster	South Korea	C	2	EB	*Ewingella americana*
KS582	King oyster	South Korea	C	1	EB	*Ewingella americana*
KS574	King oyster	South Korea	C	2	EB	*Ewingella americana*
KS588	King oyster	South Korea	C	2	EB	*Ewingella americana*
KS640	King oyster	Austria	B	2	EB	*Cedecea lapagei*
KS646	King oyster	Austria	A	2	EB	*Ewingella americana*
KS648	King oyster	Austria	B	2	EB	*Ewingella americana*
KS649	King oyster	Austria	B	1	EB	*Citrobacter freundii*
KS654	King oyster	South Korea	C	2	EB	*Ewingella americana*

KS701	King oyster	South Korea	C	2	EB	*Ewingella americana*
KS658	King oyster	Austria	B	2	EB	*Ewingella americana*
KS705	King oyster	Austria	B	2	EB	*Ewingella americana*
SH666	Shiitake	Germany	G	2	EB	*Ewingella americana*
KS680	King oyster	Austria	B	1	EB	*Ewingella americana*
KS710	King oyster	Austria	B	2	EB	*Ewingella americana*
SH688	Shiitake	Germany	G	2	EB	*Ewingella americana*
AU692	Oyster	Austria	F	2	EB	*Ewingella americana*
KS684	King oyster	South Korea	C	1	EB	*Pantoea beijingensis*
KS685	King oyster	South Korea	C	1	EB	*Ewingella americana*
KS712	King oyster	South Korea	C	2	EB	*Ewingella americana*
KS708	King oyster	South Korea	C	2	EB	*Ewingella americana*
KS714	King oyster	South Korea	C	2	EB	*Ewingella americana*
AU749	Oyster	Poland	C	1	EB	*Ewingella americana*
AU741	Oyster	Poland	C	2	EB	*Ewingella americana*
KS753	King oyster	South Korea	C	2	EB	*Ewingella americana*
KS759	King oyster	South Korea	C	2	EB	*Ewingella americana*
KS765	King oyster	South Korea	C	2	EB	*Ewingella americana*
KS743	King oyster	Austria	B	2	EB	*Ewingella americana*
KS769	King oyster	Austria	B	2	EB	*Ewingella americana*
SH755	Shiitake	Germany	D	2	EB	*Ewingella americana*
SH779	Shiitake	Germany	D	2	EB	*Ewingella americana*
KS783	King oyster	South Korea	C	2	EB	*Cedecea davisae*
KS800	King oyster	South Korea	C	1	EB	*Ewingella americana*
KS793	King oyster	Austria	B	2	EB	*Ewingella americana*
SH809	Shiitake	Germany	D	1	EB	*Ewingella americana*
SH825	Shiitake	Germany	D	2	EB	*Ewingella americana*
SH892	Shiitake	Austria	E	2	EB	*Ewingella americana*
SH923	Shiitake	Austria	E	1	EB	*Ewingella americana*
AU914	Oyster	Germany	D	1	EB	*Ewingella americana*
SH928	Shiitake	Germany	D	2	EB	*Ewingella americana*
SH938	Shiitake	Germany	D	2	EB	*Ewingella americana*
AU932	Oyster	Austria	F	2	EB	*Ewingella americana*
AU968	Oyster	Austria	F	1	EB	*Ewingella americana*

SH977	Shiitake	Germany	D	2	EB	*Ewingella americana*
SH948	Shiitake	Germany	D	2	EB	*Ewingella americana*
AU952	Oyster	Germany	D	2	EB	*Ewingella americana*
AU989	Oyster	Austria	E	2	EB	*Ewingella americana*
AU958	Oyster	Austria	E	2	EB	*Ewingella americana*
SH1005	Shiitake	Germany	C	2	EB	*Ewingella americana*
SH1000	Shiitake	Germany	C	1	EB	*Ewingella americana*
SH1015	Shiitake	Germany	D	1	EB	*Ewingella americana*
SH1032	Shiitake	Germany	D	1	EB	*Ewingella americana*
KS25	King oyster	Austria	B	1	LAB	*Lactobacillus sakei*
KS26	King oyster	Austria	B	1	LAB	*Vagococcus salmoninarum*
KS36	King oyster	Austria	B	2	LAB	*Lactobacillus sakei*
KS452	King oyster	Austria	B	1	MOX	*Moraxella osloensis*
KS456	King oyster	Austria	B	1	LAB	*Carnobacterium maltaromaticum*
SH453	Shiitake	Austria	E	1	MOX	*Moraxella osloensis*
SH454	Shiitake	Austria	E	1	COC	*Staphylococcus epidermidis*
SH461	Shiitake	Austria	E	1	COC	*Micrococcus yunnanensis*
SH465	Shiitake	Austria	E	2	COC	*Dermacoccus nishinomiyaensis*
SH496	Shiitake	Germany	G	2	LAB	*Leuconostoc mesenteroides subsp. jonggajibkimchii*
KS580	King oyster	Austria	A	2	LAB	*Vagococcus fluvialis*
KS578	King oyster	South Korea	C	2	LAB	*Lactobacillus sakei*
AU722	Oyster	Poland	C	2	MB	*Curtobacterium plantarum*
KS763	King oyster	South Korea	C	2	BAC	*Lysinibacillus fusiformis*
SH807	Shiitake	Germany	D	2	BAC	*Paenibacillus pabuli*
AU844	Oyster	Austria	E	2	MB	*Curtobacterium oceanosedimentum*
KS858	King oyster	Austria	A	2	BAC	*Bacillus circulans*
AU896	Oyster	Germany	D	1	BAC	*Solibacillus silvestris*
AU897	Oyster	Germany	D	1	BAC	*Bacillus sinesaloumensis*
SH1043	Shiitake	Germany	D	2	MB	*Microbacterium hydrocarbonoxydans*

Abbreviations: Origin – producing country; n – number of isolates; GESS – *Pseudomonas* (*P.*) *gessardii*-SG; CORRUG – *P. corrugata*-SG; FLUOR – *P. fluorescens*-SG; FRAGI - *P. fragi*-SG; JESS – *P. jessenii*-SG; KORE – *P. koreensis*-SG; MAND – *P. mandeli*-SG; PUT – *P. putida*-Group; STRAM – *P. straminea*-Group; BAC – *Bacillaceae*; COC – Coccus; EB – *Enterobacteriaceae*; LAB – lactic acid bacteria; MB – *Microbacteriaceae*, MOX – *Moraxellaceae*
